# Fluorescent Single-Walled Carbon Nanotubes for Protein Detection

**DOI:** 10.3390/s19245403

**Published:** 2019-12-07

**Authors:** Adi Hendler-Neumark, Gili Bisker

**Affiliations:** Department of Biomedical Engineering, Faculty of Engineering, Tel-Aviv University, Tel Aviv 6997801, Israel; adihendler@tauex.tau.ac.il

**Keywords:** molecular recognition, fluorescent nanoparticles, single-walled carbon nanotubes, protein detection, nanosensors

## Abstract

Nanosensors have a central role in recent approaches to molecular recognition in applications like imaging, drug delivery systems, and phototherapy. Fluorescent nanoparticles are particularly attractive for such tasks owing to their emission signal that can serve as optical reporter for location or environmental properties. Single-walled carbon nanotubes (SWCNTs) fluoresce in the near-infrared part of the spectrum, where biological samples are relatively transparent, and they do not photobleach or blink. These unique optical properties and their biocompatibility make SWCNTs attractive for a variety of biomedical applications. Here, we review recent advancements in protein recognition using SWCNTs functionalized with either natural recognition moieties or synthetic heteropolymers. We emphasize the benefits of the versatile applicability of the SWCNT sensors in different systems ranging from single-molecule level to in-vivo sensing in whole animal models. Finally, we discuss challenges, opportunities, and future perspectives.

## 1. Introduction

Molecular recognition of DNA, small molecules, proteins, or viruses is vital across many fields of research, especially for the study of the underlying mechanism of biological processes, healthcare, agriculture, food security, and environmental sciences [1,2,3]. Nanosensors play a key role in current sensing technologies, enabling a deeper understanding of previously unstudied biological phenomena [4,5,6,7]. Recent developments of novel nanosensors offer promising approaches for improved clinical diagnostics and treatments, with increasing interest in nanomaterials-based biosensors [8,9,10,11,12,13,14,15,16,17,18]. A sensor must have two functionalities; namely, target recognition and signal-transduction, which translates the recognition into a measurable signal. For recognition, a sensor can include antibodies, aptamers, DNA sequences, molecular imprints, lectins, or synthetic moieties [19,20,21,22,23,24]. Signal transduction, on the other hand, is usually achieved by labeling with fluorescent dyes or gold nanoparticles for immunohistochemistry and other approaches, providing an optical indication of target binding [25,26,27,28,29]. The combination of recognition with signal transduction contributes to the sensitivity and selectivity of the sensor in biological environments [30,31,32]. Various nanoparticles have shown potential to be highly sensitive and selective, such as metal nanoparticles, quantum dots, nanowires, graphene, graphene quantum dots, and carbon nanotubes [8,9,11,12,30,33], such that they can bind and detect biologically relevant concentrations of a target analyte. Among the numerous nanosensor platforms, the use of carbon nanotubes as sensors for biotechnological and biomedical applications is of particular interest due to their electrical and thermal properties, mechanical durability, and the wealth of options for further functionalization, doping, and chemical modifications [34]. Carbon nanotubes can be divided into two main categories according to the number of cylindrical graphene layers; namely, single-walled carbon nanotubes (SWCNTs) and multi-walled carbon nanotubes (MWCNTs). Whereas SWCNTs comprise a single one-atom-thick graphene sheet rolled to form a cylinder with a diameter ranging from 0.7 to 3 nm, MWCNTs consist of several concentric SWCNT layers whose diameters can range from approximately 1.5 nm for double-walled carbon nanotubes [35] to 220 nm for tens of layers [34].

In this review, we focus on SWCNT nanobiosensors due to their unique physical, chemical, and optical properties [36,37,38,39]. We briefly survey the properties of SWCNTs and their various biomedical applications, and then introduce different methods for recognizing proteins using their natural substrates, such as protein receptors, protein-binding partners, antibodies, or aptamers, or using a non-biological synthetic substrate bound to the SWCNT surface. We focus on recent demonstrations of utilizing synthetic recognition sites on the nanotubes to detect different proteins. The proteins do not necessarily have any affinity to the synthetic substrate, but rather to its pinned configuration when wrapped around the SWCNT scaffold. Finally, we compare SWCNT sensors and other sensing platforms, and provide a perspective on future directions.

## 2. Single-Walled Carbon Nanotubes

### 2.1. SWCNTs Properties

**Figure 1:**
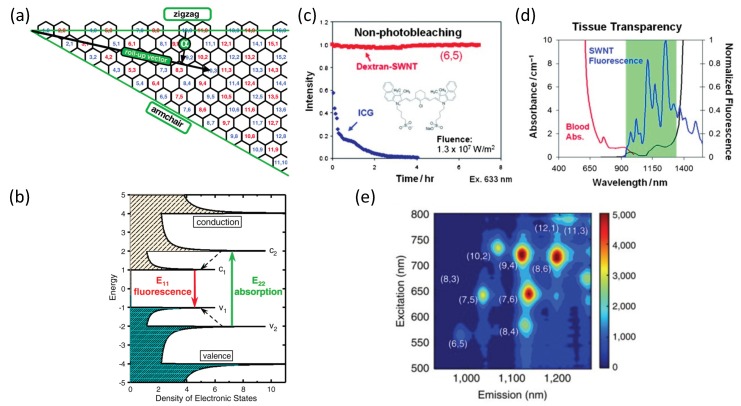
Single-walled carbon nanotubes (SWCNTs) properties. (**a**) A graphene sheet segment with indexed lattice points. A nanotube with a chiral index (n,m) is obtained by rolling the sheet along a roll-up vector originating at (0,0) up to (n,m). The chiral angle α (from 0 to 30°) is measured between the roll-up vector and the horizontal zigzag axis; the tube circumference is the length of the roll-up vector. Nanotubes with chiral indexes for which mod (n-m,3) = 0 are metallic, whereas the rest are semiconducting. (**b**) The density of electronic states of a semiconducting single-walled carbon nanotube structure. Solid arrows depict the excitation and emission transitions of interest; dashed arrows denote nonradiative relaxation. (**c**) Most fluorophores, such as indocyanine green (ICG), undergo rapid photobleaching upon continuous illumination (blue). SWCNT emission (red) remains photostable even under high fluence irradiation (1.3 × 10^7^ W m^−2^). (**d**) SWCNTs fluoresce (blue) primarily in the near–infrared regime (900–1600 nm), where blood (red) and water (black) absorbance is minimal. The figure includes tissue data adapted from Wray et al. [87], reprinted with permission from Boghossian et al. [16], and used with permission from Wiley publication. (**e**) Excitation–emission profile of polymer-functionalized SWCNT suspension. Reprinted with permission from Bisker et al. [89] and used with permission from Nature Communications.

Single-walled carbon nanotubes are one-atom-thick graphene sheets rolled to form a cylinder with a specific chirality and dimension [6] that determine their physical, chemical, electronic, and optical properties [6,18,30,40,41] (Figure 1a,b). The roll up vector, which connects two lattice points on the sp^2^ hybridized graphene sheet, ends up as the circumference of the SWCNT and defines the orientation of the honeycomb lattice of the nanotube. Larger diameter nanotubes have high persistence length [42], and smaller level spacings in their electronic density of states [43] which in turn affect the optical transitions [44]. The lattice structure further determines the chemical interaction of the SWCNT with adsorbed surfactants or polymers, thereby enabling chirality-based separation and sorting [45,46,47].

Having a diameter of the order of 1 nm, and length in the range of 100 nm up to several micrometers, SWCNTs are one-dimensional, high-aspect-ratio nanocarbon materials, with high surface areas that can be readily functionalized. Without surface functionalization, SWCNTs are hydrophobic and tend to bundle due to strong van der Waals attraction forces [48]. In order to form a colloidal suspension of individually dispersed SWCNTs, they are usually non-covalently functionalized with amphiphilic molecules or polymers by sonication [18,48,49,50,51,52]. Proper surface functionalization can render them biocompatible, and thus, suitable for numerous biomedical applications, including sensing, drug delivery, nanoinjection, phototherapy, imaging, or artificial actuation [13,30,51,53,54,55,56,57,58,59,60,61,62,63,64,65,66,67,68,69,70,71,72,73].

The high surface-to-volume ratio can facilitate a relatively large cargo load on SWCNTs for efficient delivery applications. For example, SWCNTs can function as a universal drug delivery system (DDS) for small interfering RNA (siRNA) and other oligonucleotides, having circulation times ranging from minutes to hours. The delivery of siRNA has been observed to include pharmacokinetics, toxicity, antitumor activity, and target protein knockdown in several cell lines [74]. In addition, SWCNTs can penetrate cells and release siRNA into the cytoplasm [49], which is of great importance for gene-silencing applications. Moreover, recent studies have reported the utilization of carbon nanotubes for unassisted delivery of plasmid DNA and siRNA into a variety of model and non-model plant species [75,76,77].

Semiconducting SWCNTs have unique optical properties, including bright fluorescence emission in the near-infrared (nIR) spectral range mainly between 900 and 1600 nm, and a broad absorption spectrum compared to organic molecules [78]. In addition, they do not photobleach or blink [16] (Figure 1c). The photostable nIR fluorescence, along with robust functionalization, allow for the prolonged detection of SWCNTs through biological samples such as tissues, blood, and cells, as they are relatively transparent in this spectral range [15,30,41,51,69,73,79,80,81,82,83,84,85,86] (Figure 1d). Human blood, for instance, has a narrow optical transparency window from 900 to 1400 nm where light can penetrate to approximately 3–5 cm [87]. Only a few conventional markers absorb or emit strongly in this region; however, some suffer from low photochemical stability or poor biocompatibility [51,88]. In addition to the optical properties, the physical dimensions of SWCNTs in the order of nanometers to a few microns match the typical size of biological molecules, enabling precise targeting and visualization [30]. Thus, SWCNTs are attractive candidates for biomedical imaging, detection, and sensing applications.

### 2.2. SWCNTs as Optical Sensors

The fluorescence signal of SWCNTs is sensitive to the environment and can be affected by global changes in pH and ionic strength [90] or local changes in surface functionalization or even single-molecule adsorption [91]. The surface functionalization forms a corona phase surrounding the nanotube scaffold, which mediates the interaction of the SWCNT with molecular analytes in its proximity, and thus determines the fluorescence modulation upon surface binding. The SWCNT fluorescence originates from a radiative recombination of excitons, which have strong binding energy [16]. Upon target binding, there are several mechanisms that can lead to the modulation of the emitted light, including exciton quenching due to competitive non-radiative decay, a shift in the Fermi level leading to absorption bleaching, and reorientations of the solvent dipole moments in close proximity to the SWCNT due to conformational changes of the corona phase, resulting in a solvatochromic shift [92,93].

Hence, SWCNTs can be used for sensing applications as fluorescence signal transducers, with the benefits of high photostability, lack of photobleaching, and physical size comparable to the typical size of target biomolecules [30,49]. The various chiralities can enable multiplexed detection by monitoring the emission in different wavelength channels (Figure 1e), facilitating high throughput screening [6] and hyperspectral imaging [94]. Further, different chiralities within the same SWCNT suspension can respond differently to a target analyte, owing to differences in the chemical interactions between the wrapping polymer and the underlying lattice structure of the nanotube [95].

Owing to their unique optical properties, SWCNTs have been utilized as optical sensors for biomarkers of human diseases, including different types of cancer, glucose levels in diabetics, and H_2_O_2_ in reactive oxygen signaling pathways [83,96,97,98]. Single-walled carbon nanotubes functionalized with nucleic acids or peptides form stable complexes, even in complex biological environments [99,100,101,102,103,104,105], with increased thermal stability up to 200 °C [105]. Moreover, SWCNTs functionalized with DNA sequences containing an endonuclease recognition site have been successfully used to study restriction enzyme activity by monitoring their fluorescent emissions [106]. The DNA-SWCNTs have shown increased fluorescence intensity in response to neurotransmitters and have successfully detected dopamine efflux in neuroprogenitor cell cultures [107,108,109,110,111] and in acute brain slices [112,113]. Further, (GT)_6_-SWCNT has successfully detected dopamine and norepinephrine in a broad range of pH and salt concentrations, suggesting the potential compatibility for in-vivo neurophysiological use [113,114]. A recent study has demonstrated the recognition of the neurotransmitter serotonin using SWCNTs wrapped with a serotonin-aptamer. This nanosensor was immobilized on a glass surface, on which human blood platelets were cultured, and were shown to detect serotonin release patterns from the cells in real time [115]. Additionally, DNA-wrapped SWCNTs were utilized for the detection of a single-stranded RNA genome of an intact HIV particle [116] and of doxorubicin, a chemotherapy drug effective against dividing cells due to its affinity to DNA [117]. Further, DNA-SWCNTs were engineered to quantify microRNA hybridization, by a solvatochromic-like response following DNA displacement from the nanotubes’ surface [118]. In addition, SWCNTs functionalized with boronic acid-modified dextran, PEG-brush, and rhodamine isothiocyanate functionalized-PEG were shown to be selective sensors for the small molecules riboflavin, L-thyroxine, and estradiol, respectively [119].

## 3. Protein Recognition with SWCNTs

The recognition of large bio-macromolecules poses a different challenge owing to the size, complexity, and various conformations of the target, as in the case of proteins [120]. Nevertheless, SWCNTs have been successfully utilized for protein detection and for the study of protein–protein interaction by surface functionalization with either natural substrates or synthetic ones.

### 3.1. Natural Protein Recognition

One approach for protein detection is to use the natural binding partner of the target protein as a recognition site on the SWCNTs. This can be achieved, for example, by using an antibody, an aptamer, or a DNA recognition sequence, in order to exploit the original protein–protein or protein–DNA interactions for sensing applications.

**Figure 2:**
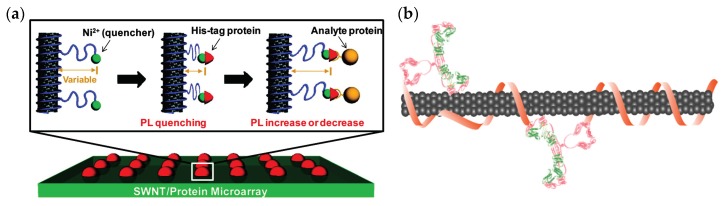
Detecting protein–protein interactions using SWCNTs. (**a**) Schematic of label-free protein sensor array with fluorescent SWCNTs. The SWCNT suspension was spotted on a glass and functionalized with NTA-Ni^2+^ to bind his-tagged capture proteins and detect the interaction between the captured protein and a target protein. The his-tagged capture proteins were first immobilized by the NTA-Ni^2+^ groups through their his-tag residues. Subsequently, upon the addition of a target protein to each spot and their binding to the corresponding capture proteins, the distance between the Ni^2+^ quencher and the SWCNT surface changed, resulting in a fluorescence modulation. Reprinted with permission from Ahn et al. [121], copyright 2011 American Chemical Society. (**b**) Illustration of the anti-uPA–DNA–SWCNT complexes. Reprinted with permission from Williams et al. [124]. Copyright 2018 American Chemical Society.

A label-free detection was demonstrated in Ahn et al. [121] using nanotubes functionalized with chitosan polymer modified with nitrilotriacetic acid (NTA) chelator. The chitosan was utilized owing to the accessibility of functional groups for additional modification. The NTA chelated Ni^2+^ and served as a proximity quencher modulating the SWCNT fluorescence intensity as a function of distance (Figure 2a). The NTA-Ni^2+^ group can bind to any hexahistidine tagged (his-tag) capture protein, which serves as a natural binding site for the protein of interest. For example, a his-tagged protein A bound to the NTA-Ni^2+^ group was used to capture human immunoglobulin G (IgG) [122]. A binding of the target protein leads to a modulation of the fluorescence intensity, enabling the studying of protein–protein interactions, protein glycoprofiles, and protein quantification [30,121,122,123].

Satishkumar et al. [125] used fluorescent SWCNT sensors for the detection of avidin by conjugating redox-active dyes bound to a recognition element, biotin, to the SWCNT surface. The biotinylated dyes were quenched when adsorbed onto the SWCNTs, such that avidin binding resulted in their desorption from the nanotubes and the recovery of the fluorescence. The mechanism of the fluorescence quenching relies on oxidative charge-transfer reactions with small redox-active organic dye molecules [30]. This concept, of dye-ligand complex conjugated to SWCNT, can be highly versatile for a wide range of bioanalytes, through the choice of the specific receptor group attached to the quenched dye [125].

Additional studies showed the detection of the prostate cancer biomarker, urokinase plasminogen activator (uPA), using DNA-SWCNTs conjugated to an anti-uPA antibody (Figure 2b) [124], and the detection of single RAP1 proteins secreted from individual *Escherichia coli* cells using SWCNTs functionalized with the RAP1 aptamer [126]. Further, Lee et al. demonstrated the optical detection of insulin and platelet-derived growth factor with the corresponding aptamers through two distinct mechanisms; namely, direct protein binding to the aptamer-SWCNT complex and the detachment of the aptamer from the SWCNTs’ surface following protein binding, both leading to a decrease in fluorescence intensity [127]. Moreover, SWCNTs functionalized with HE4 antibody showed a nanomolar sensitivity for HE4, a biomarker for high-grade ovarian carcinoma, enabling noninvasive optical detection of cancer biomarkers [128].

### 3.2. Synthetic Protein Recognition

**Figure 3:**
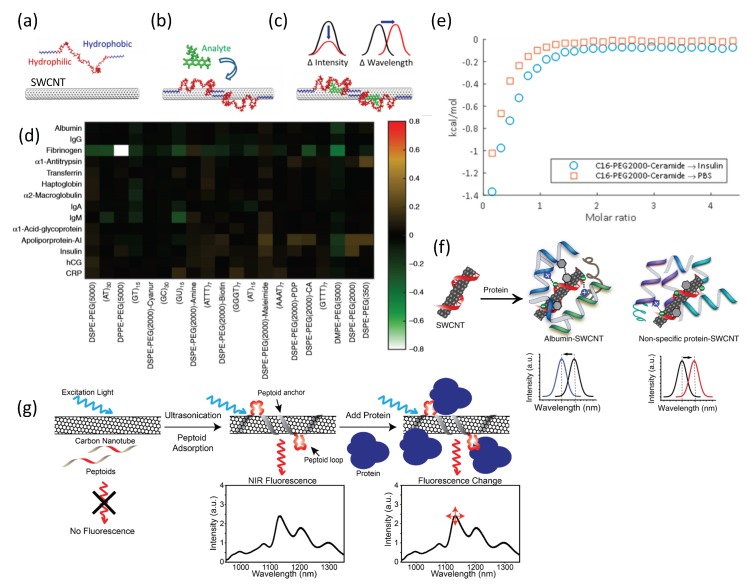
Synthetic coronae for protein recognition. (**a**) SWCNT is suspended using a synthetic heteropolymer with hydrophilic and hydrophobic domains. (**b**) The heteropolymer is adsorbed onto the surface of the SWCNT to form a corona phase around the nanotube, which facilitates the recognition of a specific analyte, (**c**) resulting in a fluorescence modulation signature for sensing applications. From Landry et al. [134], used with permission from Sensors. (**d**) Heat map of the normalized response of the SWCNTs’ fluorescence intensity to the various proteins demonstrating the selective and specific response of (DPPE)-PEG(5kDa)-SWCNT to fibrinogen. From Bisker et al. [89], used with permission from Nature Communications. (**e**) Binding isotherm for the titration of C_16_-PEG(2kDa)-ceramide into insulin solution (blue circles) or PBS (red squares) plotted against the molar ratio of C_16_-PEG(2kDa)-ceramide to insulin. The overlapping curves of the injections into insulin or PBS indicate that the heat released in both cases is similar, manifesting a lack of affinity between insulin and C_16_-PEG(2kDa)-ceramide without the nanotube scaffold. Reprinted with permission from Bisker et al. [129]. Copyright 2018 American Chemical Society. (**f**) A proposed albumin recognition model by the carboxylate-rich, hydrophobic polymer, potentially due to mimicking the head group of fatty acids that bind albumin through salt bridges or hydrogen bonds. Reprinted from Budhathoki-Uprety et al. [145], used with permission from Nature Communications. (**g**) Peptoid-SWCNT complexes for protein recognition. An anchor region of the peptoid is adsorbed to the SWCNT surface, whereas a flanking loop segment interacts with the target protein, resulting in fluorescence modulation. Reprinted from Chio et al. [146]. Copyright 2019 American Chemical Society.

Molecular recognition can also be achieved using a synthetic SWCNT corona [119]. In this approach, a synthetic amphiphilic polymer is adsorbed onto the hydrophobic surface of the SWCNT. The hydrophobic domains of the polymer form a stable polymer-nanotube complex, whereas the hydrophilic regions extend into the aqueous environment (Figure 3a,b). The conformation of the amphiphilic polymer, that is, the corona phase, can enable the binding of a specific analyte, resulting in spectral modulations (Figure 3c). In order to discover new corona phases for molecular recognition, a library of polymer-conjugated SWCNT is screened against a panel of analyses, and the nIR fluorescence emission is monitored for intensity changes or wavelength shifts [89,119,129,130]. A successful screen results in a corona phase that can specifically and selectively recognize a target analyte [119]. In parallel, theoretical efforts have led to preliminary design principles of a specific configuration of a short polymer wrapping that would recognize the contour and functional groups of a small molecule or a protein of interest [131].

The interaction between the target analyte and the functionalized SWCNTs, and the resulting fluorescence modulation, depend on many factors, including the nanotube chirality, the composition and valency of the polymer corona, and the lipophilicity and redox potential of the target [60,95,107,122,132]. The molecular interaction mediated by the SWCNT corona is an active area of research, where experimental and numerical tools are rapidly being developed in order to shed light on the underlying mechanism of this complex interaction [95,131,132,133,134,135].

The first high-throughput screening of synthetic polymer coronae of SWCNTs targeted a small molecules library [119]. Subsequently, the first protein-targeted corona phase screen has led to the discovery of a sensor for the protein fibrinogen [89]. In this study, 20 SWCNT corona phases were screened against a protein library consisting of 14 proteins from the whole human blood, either the most abundant or of clinical significance, including albumin, transferrin, haptoglobin, fibrinogen, α_1_-antitrypsin, α_1_-acid-glycoprotein, human chorionic gonadotropin (hCG), α_2_-macroglobin, immunoglobulin A (IgA), IgG, IgM, apolipoprotein A-I, C-reactive protein (CRP), and insulin [89]. The screen revealed a specific sensor for fibrinogen, using dipalmitoyl-phosphatidylethanolamine (DPPE)-PEG(5kDa) corona (Figure 3d). Fibrinogen is one of the most abundant proteins in the plasma, with an elongated structure that consists of three globular domains connected by coiled-coil helical chains [136,137]. The detection of fibrinogen was also demonstrated in competitive assays in the presence of albumin, which is usually used as a nonspecific binding agent [138,139,140], or in serum environment. Nonselective parameters such as the molecular weight and hydrophobicity of the proteins, or the surface coverage of the polymer, showed no correlation with the fluorescence response, supporting the hypothesis that the combination of the three-dimensional structure of a target protein, along with the conformation of the phospholipid-PEG corona adopted when pinned around the nanotubes, is a key factor in successful molecular recognition [89].

An extended corona phase screen against the same protein panel revealed a sensor for insulin [129]. Insulin is a small peptide hormone which plays a key role in blood glucose regulation [141]. Through the secretion of insulin, the pancreas stimulates glucose uptake in order to synthesize lipids, and inhibits the production of ketone bodies and the breakdown of proteins, glycogen, and lipids [142]. The high-throughput corona phase screen was done with PEGylated-lipids-SWCNTs, where the C_16_-PEG(2kDa)-ceramide-SWCNT complex showed a specific and selective quenching response to insulin. The corona phase showed no prior affinity towards insulin, validated using isothermal titration calorimetry (Figure 3e), by comparing the heat released while injecting the C_16_-PEG(2kDa)-ceramide into an insulin solution or phosphate-buffered saline (PBS) [129]. The new synthetic nIR fluorescent nanoparticle paves the way to real-time detection of insulin levels in vivo using an encapsulating implant [30,55,62,83,143,144]. Inference of insulin levels in the various body compartments can be achieved using a pharmacokinetic model of insulin, glucose, and glucagon metabolism [55].

In a recent study, Budhathoki-Uprety et al. developed an albumin nanosensor using SWCNTs functionalized with polycarbodiimide polymers incorporating phenyl rings, which mimic fatty acid binding to albumin [145]. Albumin detection was demonstrated in minimally processed urine samples of microalbuminuria patients under ambient conditions, with similar sensitivity compared to antibody-based clinical assay, suggesting that this antibody-free detection can facilitate diagnosis in point-of-care and resource-limited settings (Figure 3f) [145]. A different approach for protein recognition was demonstrated by Chio et al. using peptoid-functionalized SWCNTs [146]. Peptoids are easy to manufacture, resistant to proteases activities, and can specifically recognize enzymes and proteins [147,148]. In their study, Chio et al. utilized an anchor-loop peptoid corona for the recognition of the lectin protein wheat germ agglutinin (WGA), and further validated that the WGA kept its functionality of binding to target sugars (Figure 3g) [146].

## 4. SWCNTs Advantages

Traditional recognition methods use antibodies to identify small and macromolecular targets. Antibody-antigen pairs have a wide range of applications, from diagnostics and therapeutics to basic and clinical research [149,150]. Although they benefit from high selectivity and specificity to the antigens, a major limitation of antibodies in high-throughput research is the need for injecting the antigen into an animal as the first step of production [151].

Protein corona phase molecular recognition using SWCNT offers an alternative approach for various assays, in which degradation, stability, cost, and production scale prevent natural recognition elements, such as antibodies, from being employed. In this method, the synthetic polymer used for recognition does not necessarily have any prior affinity to the target protein; rather, its pinned configuration upon wrapping the nanotube surface forms a conformational binding site. A discovery of such nanosensors can be driven by high-throughput screening with rapid manufacturing processes [119]. This can generate synthetic, non-biological antibody analogs that can overcome some of the limitations of the conventional ones, including long development times; high production costs; the need for living organisms for initial production; challenging reproducibility; poor stability due to hydrolysis in ambient temperature, resulting in limited shelf time’ and sensitivity to degradation while circulating in vivo [152,153,154]. In contrast, SWCNTs demonstrated long-term stability in vivo [62] and were shown to protect DNA or siRNA from cellular nuclease degradation [49,155]. Hence, SWCNT recognition offers a stable and reproducible construct that can push forward discovery research in the field [18,89,119].

The fluorescence of SWCNTs has several advantages over common organic fluorescent dyes and fluorescent quantum dots. The main limitation of organic fluorophores is the inevitable photobleaching that restricts their utilization for real-time microscopy experiments lasting several hours [51,156]. Further, organic fluorophores are quenched when jointly applied with hematoxylin and eosin (H&E), an important stain used for evaluation of histological sections [157,158,159]. Quantum dots are attractive probes for microscopy and imaging, owing to their photophysical properties, including their photostability and narrow bandwidth fluorescence emission with a wide excitation range [160]. They are mainly used as inert markers [161,162,163,164,165,166] and FRET-based sensors [167] for both in vitro and in vivo applications. They are highly luminescent semiconducting nanoparticles, and are approximately 100 times more resistant to photobleaching than organic fluorophore. Nevertheless, they suffer from signal attenuation under prolonged excitation, fluorescence blinking, complicated surface chemistry, and potentially cause long-term heavy-metal toxicity [168,169,170,171]. Single-walled carbon nanotubes overcome these limitations owing to their inherent non-photobleaching, non-blinking fluorescence, and their sp^2^ hybridized all-carbon structure that gives rise to easy surface functionalization and biocompatibility [14,62,172]. Hence, SWCNTs are subjected to intensive research in many emerging applications of optical nanosensors that exploit their nIR fluorescent emission and surface chemistry for target recognition and signal transduction [10,16,30,41,50].

## 5. Conclusions

This review provides an overview and a comprehensive survey of the utilization of SWCNTs for biosensing applications. The non-photobleaching, non-blinking fluorescent emission of SWCNTs plays a key role in rendering them optical sensors, enabling in situ, label-free, real-time detection with both spatial and temporal resolution [89,113,115]. Recent studies have demonstrated the detection of proteins using various approaches for surface functionalization, including natural substrates [53,121,125] and synthetic polymers [89,129,145], with the potential to enable long-term continuous monitoring of important biomarkers or to replace costly and time-consuming laboratory testing [173]. We have highlighted the advantages of SWCNTs for in-vivo and in-vitro biomedical applications such as drug delivery, imaging, and sensing, focusing on protein recognition. Their considerable potential to advance research and applications in this field has drawn increasing attention in recent years, opening new avenues for future discoveries [18].

In summary, the unique properties of SWCNTs make them excellent candidates for sensing proteins and bio-macromolecules, with optical signal transduction, where advancements in nanotechnology design, synthesis, characterization, and modeling will continue to push forward the discovery of new SWCNT-based fluorescent sensors.
